# Integrative Models of Brain Structure and Dynamics: Concepts, Challenges, and Methods

**DOI:** 10.3389/fnins.2021.752332

**Published:** 2021-10-29

**Authors:** Siva Venkadesh, John Darrell Van Horn

**Affiliations:** ^1^Department of Psychology, University of Virginia, Charlottesville, VA, United States; ^2^School of Data Science, University of Virginia, Charlottesville, VA, United States

**Keywords:** neuroimaging, network dynamics, multi-modal connectivity, neuroanatomy, structure-dynamic coupling

## Abstract

The anatomical architecture of the brain constrains the dynamics of interactions between various regions. On a microscopic scale, neural plasticity regulates the connections between individual neurons. This microstructural adaptation facilitates coordinated dynamics of populations of neurons (mesoscopic scale) and brain regions (macroscopic scale). However, the mechanisms acting on multiple timescales that govern the reciprocal relationship between neural network structure and its intrinsic dynamics are not well understood. Studies empirically investigating such relationships on the whole-brain level rely on macroscopic measurements of structural and functional connectivity estimated from various neuroimaging modalities such as Diffusion-weighted Magnetic Resonance Imaging (dMRI), Electroencephalography (EEG), Magnetoencephalography (MEG), and functional Magnetic Resonance Imaging (fMRI). dMRI measures the anisotropy of water diffusion along axonal fibers, from which structural connections are estimated. EEG and MEG signals measure electrical activity and magnetic fields induced by the electrical activity, respectively, from various brain regions with a high temporal resolution (but limited spatial coverage), whereas fMRI measures regional activations indirectly via blood oxygen level-dependent (BOLD) signals with a high spatial resolution (but limited temporal resolution). There are several studies in the neuroimaging literature reporting statistical associations between macroscopic structural and functional connectivity. On the other hand, models of large-scale oscillatory dynamics conditioned on network structure (such as the one estimated from dMRI connectivity) provide a platform to probe into the structure-dynamics relationship at the mesoscopic level. Such investigations promise to uncover the theoretical underpinnings of the interplay between network structure and dynamics and could be complementary to the macroscopic level inquiries. In this article, we review theoretical and empirical studies that attempt to elucidate the coupling between brain structure and dynamics. Special attention is given to various clinically relevant dimensions of brain connectivity such as the topological features and neural synchronization, and their applicability for a given modality, spatial or temporal scale of analysis is discussed. Our review provides a summary of the progress made along this line of research and identifies challenges and promising future directions for multi-modal neuroimaging analyses.

## Introduction

The structural and the dynamical complexities of mammalian brains necessitate multi-modal and multi-scale analyses. Understanding how the neural network dynamics emerge from, and their optimal ranges are constrained by the underlying structure is an important goal in brain sciences.

Brain regions consist of networks of neurons with remarkably diverse structural and oscillatory profiles (Wheeler et al., 2015; Komendantov et al., 2019). An isolated neuron’s activation patterns are largely determined by the distributions of various ion channels and their conductance densities along the membrane (Druckmann et al., 2007) and relatively independent of the neuron’s precise morphological features (Markram et al., 2015). Furthermore, similar neuronal dynamics can result from various combinations of ionic conductances distributed along the neuronal structure, underscoring a many-to-one relationship between structure and dynamics at the level of individual neurons (Schulz et al., 2006; Marder, 2011; Rathour and Narayanan, 2014).

Neural plasticity is a hallmark of brain circuits. Plasticity in the intrinsic ionic conductance of neurons and the coupling conductance of synapses synergistically interact to achieve optimal coordination in neural networks (Maffei and Fontanini, 2009; Lane et al., 2016). While activity-dependent synaptic plasticity modifies the strength of existing synapses based on the relative timing of pre- and post-synaptic neuronal firing events (Hebb, 2005), other forms of activity-dependent plasticity also occur in neural networks. Neurons maintain their target activity level in a homeostatic manner by scaling existing synapses (Turrigiano et al., 1998; Magee and Cook, 2000; Rathour and Narayanan, 2014), changing dendritic spine numbers (Trachtenberg et al., 2002), forming new synapses (Knott et al., 2006; Bastrikova et al., 2008), eliminating existing ones (Bastrikova et al., 2008), and even changing the axonal branching patterns (De Paola et al., 2006). Such homeostatic regulatory mechanisms are hypothesized to drive structural changes in neural networks (Butz et al., 2009; van Ooyen and Butz-Ostendorf, 2019). The mesoscopic network architecture, which is characterized by the connectivity between diverse neuronal types (Rees et al., 2016), also likely supports the emergence of complex dynamics. Many-to-one relationship between structure and dynamics exists at this level (Marder, 2011), and it may be a characteristic feature of biological systems at multiple levels (Edelman and Gally, 2001; Friston and Price, 2003).

**FIGURE 1:**
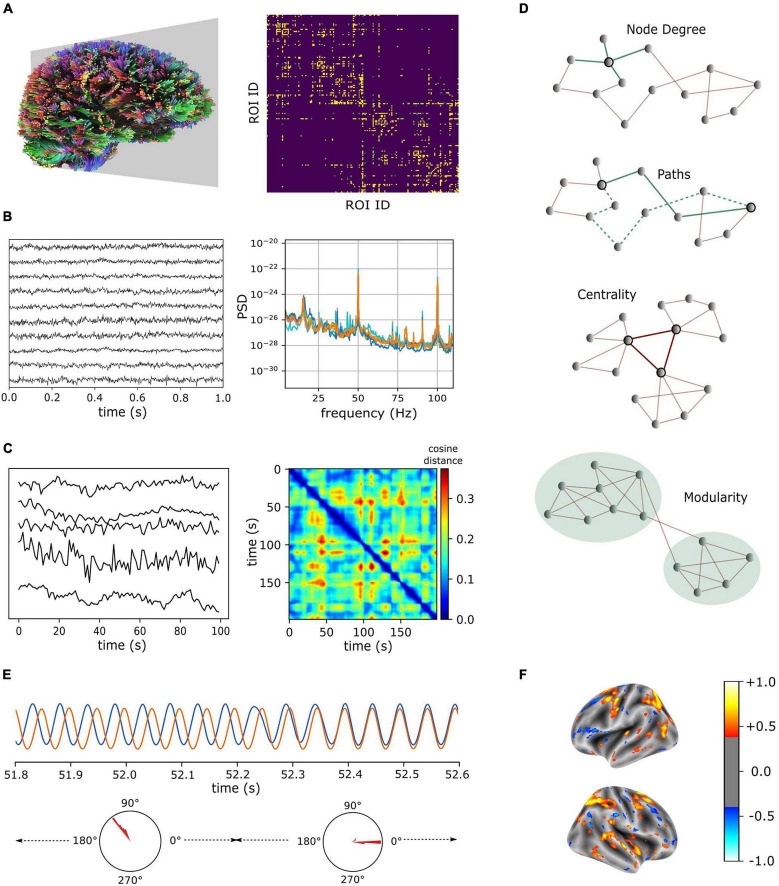
Neuroimaging modalities **(A–C)** and characteristic features **(D,E)** for brain network structure and dynamics. **(A)** Fiber tracts estimated from dMRI (left) and connectivity matrix computed for brain regions that were parcellated according to Destrieux et al. (2010). **(B)** Recordings from MEG (10 representative channels, left) and their power spectral densities (right). **(C)** Sample BOLD signals measured from resting-state fMRI (left). Time-varying vectors of (resting-state fMRI) connectivity degrees showing intermittent similarities over time (right). dMRI and MEG data were obtained from CamCAN repository (Taylor et al., 2017) and fMRI data were obtained from the connectomeDB lifespan cohort (Van Essen et al., 2013). **(D)** Features of a graph (from top to bottom): Highlighted node has a degree of 4, two representative paths between the highlighted nodes are given in green (solid green denotes the *shortest* path), central nodes in a graph form a hub, and a graph is subdivided into two communities based on the intra- and inter-community connection densities. **(E)** Metastable attractors in a system of interacting oscillators. Phase difference (mode) between two oscillators is stable over several cycles and endogenously switches between different modes. **(F)** A schema illustrating the outcome of regression analysis that uses regional characteristic features to identify predictive markers of neurological disorders (color bar denotes model coefficients).

Neuroimaging modalities including, but not limited to, Diffusion-weighted Magnetic Resonance Imaging (dMRI), Electroencephalography (EEG), Magnetoencephalography (MEG), and functional Magnetic Resonance Imaging (fMRI) enable *brain-wide* measurements of macroscopic structural connectivity and neural activations. The dMRI measures the movement of water along the axonal fibers. Tractography techniques (Mori et al., 1999; Mori and van Zijl, 2002) aim to reconstruct the trajectories of axonal projections based on the fractional anisotropy of diffusion process. However, structural connections estimated from the dMRI are undirected. EEG records the electrical activity of neuronal populations and MEG detects the magnetic fields induced by intracellular currents (Singh, 2014). EEG and MEG signals record neural activity at a high temporal resolution capturing neural oscillations in high frequency bands such as the gamma (30–80 Hz) (Figure 1B). However, their spatial coverage is limited to the number of channels used for recording, which is typically only in hundreds. fMRI, on the other hand, measures neural activity indirectly via the blood-oxygen-level-dependent (BOLD) signals, which reflect (delayed) hemodynamic responses to neural activations (Hillman, 2014). Moreover, fMRI records BOLD signals at a relatively high spatial resolution (hundreds of thousands of voxels of recordings), though hemodynamic responses are low-frequency (<0.25 Hz) convolutions of neural oscillations (Figure 1C).

The last decade has seen several large-scale brain data acquisition efforts (Alivisatos et al., 2012; Koch and Reid, 2012; Markram, 2012; Van Essen et al., 2013). However, a relative lack of progress toward conceptual understanding in the brain sciences is also recognized (Frégnac, 2017). Theoretical investigations of the relationship between network structure and emergent dynamics are valuable, and they promise to fill the gap in our empirical knowledge of the multi-scale mechanisms in the brain. In this article, we first describe a few theoretical frameworks to interpret empirically measured structural connectivity and functional patterns in the brain. Then, we describe current knowledge of the macroscopic brain structure-dynamics relationships. Finally, we discuss methods that focus on a mechanistic understanding of the coupling between brain structure and dynamics with a focus on the brain-wide network models of spontaneous dynamics.

## Theoretical Frameworks to Interpret Empirically Measured Brain Connectivity

Several network analysis techniques are used to characterize brain connectivity estimated from neuroimages. Representing neuroimaging data as a connectivity matrix is usually a necessary step before many of these techniques can be employed (Figure 1A). The connectivity matrix defines pairwise relationships such as the strengths of the anatomical connections and correlations in activations between brain regions (following the terminology used in the literature, pair-wise regional correlations in activations between brain regions will be referred to as functional connectivity). In this section, we briefly summarize some of the widely used techniques and promising theoretical frameworks to quantitatively characterize brain structure and dynamics. We limit our discussion to the graph theoretic metrics commonly applied in neuroimaging and the frameworks which emphasize synergistic dynamics in complex systems.

### Characteristic Measures of Network Connectivity

A *graph* is a data structure that represents a network as a set of nodes (vertices) and links (edges). The nodes and links correspond to the brain regions and connections between them, respectively, when the whole-brain structural neuroimage is represented using a graph data structure. The entries of connectivity matrix could be either binary, indicating the presence/absence of connections between a pair of regions, or real numbers indicating the strength of connections between regions. It is worth pointing out that *semantic networks* were recently introduced to represent connectomes as a knowledgebase of brain regions that enable combining multimodal information (Kopetzky and Butz-Ostendorf, 2018; Chen et al., 2021).

Representing the anatomical connectivity as a graph data structure enables characterization of its global and local properties using simple metrics. For example, *node degree* is a local property, and it denotes the number of links connected to the node (Figure 1D). Node degree is also referred to as degree centrality (see next paragraph for other centrality measures). At the core of several graph metrics is the notion of “shortest paths” between nodes. A path is a sequence of unique nodes visited by following their links (Figure 1D). Identification of the shortest path between two nodes in a graph (i.e., a path that consists of minimum number of visited nodes) is accomplished through heuristic optimization algorithms. The average of the shortest path lengths between all pairs of nodes in a graph is termed as *characteristic path length*. The average of inverse shortest path lengths is the *global efficiency* of a graph, and it is widely used to characterize the integration of different brain regions. Similarly, the *local efficiency* is the average of global efficiencies computed on the subgraphs consisting only of the immediate neighbors of nodes. The local efficiency is used to characterize the segregation of nodes in the graph. The global efficiency of the structural human brain connectivity increases with age (Hagmann et al., 2010) suggesting an increase in network integration and/or a decrease in local segregation in aging brains (Dennis et al., 2013a). Such path-based metrics have also been applied to functional connectivity, and reductions in functional segregation have been reported with increasing age (Chan et al., 2014; Geerligs et al., 2015). However, findings from other functional connectivity studies are inconsistent with these reports (Achard and Bullmore, 2007; Sala-Llonch et al., 2014). It is worth mentioning that applicability of path-based metrics for functional connectivity is questionable (Rubinov and Sporns, 2010). Here, a functional path is merely a sequence of statistically correlated brain regions, and it is unclear whether it truly reflects the efficiency of “information transfer/integration,” as it is often interpreted.

*Betweenness centrality* of a node is the fraction of all shortest paths in the graph that visit the given node. Similarly, *rich-club* coefficient quantifies the extent to which high-degree nodes are also connected to each other (van den Heuvel and Sporns, 2011). Brain regions with high centrality measures are often referred to as hubs (Figure 1D), and they may be crucial in coordinating functions of different brain regions (Schmidt et al., 2015). Betweenness centrality and rich-club measures are closely related, as 89% of all shortest paths visit one or more of rich-club nodes in the human brain (van den Heuvel and Sporns, 2011). However, a systematic network lesioning study revealed a core scaffold of anatomical connections that are crucial to the integrative properties of the human brain, and they were found to be distinct from the connections between rich-club nodes in the brain (Irimia and Van Horn, 2014). Similarly, *eigenvector centrality* (Bonacich, 2007) measures a node’s influence on the network. It favors nodes that are connected to other nodes with high eigenvector centrality. Eigenvector centrality is also applicable for the analysis of functional connectivity (Lohmann et al., 2010). Both degree centrality and eigenvector centrality measures were shown to be related to altered resting-state network connectivity in individuals with type I diabetes (van Duinkerken et al., 2017). However, in individuals with subjective memory complaints, degree centrality was altered in bilateral hippocampus, left fusiform and inferior parietal regions, and no changes in eigenvector centrality was reported (Li et al., 2018). The rich-club organization of the anatomical connectivity changes during development and the rich-club coefficient increases with age (Dennis et al., 2013b). The rich-club organization is also altered in pathological conditions including Schizophrenia (van den Heuvel et al., 2013), Alzheimer’s (Yan et al., 2018) and Parkinson’s diseases (Liu et al., 2021).

*Modularity* is a measure that quantifies the extent to which a network may be subdivided into non-overlapping groups or communities (Figure 1D). A number of algorithms exist to identify community structures in large networks (Pons and Latapy, 2005; Newman, 2006; Reichardt and Bornholdt, 2006; Rosvall and Bergstrom, 2007; Blondel et al., 2008), and the method proposed by Blondel et al. (2008) is one of the most widely used algorithms. It uses an optimization heuristic that maximizes the number of within-community links and minimizes the number of across-community links. Since many community detection algorithms rely on greedy optimization, repeated stochastic trials are necessary to robustly identify community structures in large networks. This is because different stochastic trials may identify distinct community structures that are equally (but only locally) optimal for a given heuristic metric. Therefore, optimization solutions from multiple trials should be aggregated to derive consensus communities (Sporns, 2018). Metrics such as Rand index (Rand, 1971), which quantifies the similarity between clusters, can be employed to evaluate the solutions of modularity optimization (Betzel et al., 2016). Another important consideration is that modularity detection has a resolution limit, which depends on the number of connections in the network, and community structures smaller than this resolution might not be resolved (Fortunato and Barthélemy, 2007). Performing modularity analysis across multiple resolutions and subsequently identifying consensus communities from hierarchically modular structures can address this limitation (Jeub et al., 2018; Sporns, 2018). Modularity analysis is also applicable to functional connectivity, however, the dynamical nature of modular organizations in functional connectivity should be noted (Betzel et al., 2016). Reductions in modularity with increasing age have been consistently reported (Onoda and Yamaguchi, 2013; Cao et al., 2014; Geerligs et al., 2015) suggesting reductions in functional segregation. This age-related reduction was also reported to be more pronounced in individuals on the Autism Spectrum (Henry et al., 2018). Modularity in resting-state networks was shown to be reduced in Alzheimer’s disease due to increased coupling between frontoparietal and default-mode networks (Contreras et al., 2019). Finally, the modularity metric was reported to be negatively correlated with cognitive performance in Parkinson’s disease (PD) patients with mild cognitive impairment (Baggio et al., 2014).

### Frameworks to Characterize Network Dynamics

Description of functional connectivity that emphasizes temporal evolution and collective dynamics maybe more meaningful than the spatialized descriptions based on path-based graph measures. Here, we briefly review the theoretical frameworks of metastability, self-organized criticality and integrated information theory, all of which emphasize synergistic transitory dynamics in systems of interacting elements.

Metastability is a conceptual framework that characterizes the temporal evolution of a system in terms of its integration and segregation tendencies (Tognoli and Kelso, 2014). In a metastable system, the elements show transiently fixed relationship with each other (e.g., transiently synchronized activations of neural populations in the brain). Coexistence of synchronized and desynchronized behaviors in a system of interacting oscillators is an indication of its metastable nature (Shanahan, 2010; Kasatkin et al., 2019). Importantly, such behaviors are short-lived, and the system endogenously transitions between different attractor (synchronized) states, whose basins of attractions can be arbitrarily close in the phase space (Tsuda, 2009). Metastability is believed to be a necessary physical property underlying the coordinated dynamics of spatially distributed neuronal populations in the brain (Fingelkurts and Fingelkurts, 2004; Freeman and Holmes, 2005). Metastability in a system of interacting oscillators can be quantified by using the oscillators’ instantaneous phases as variables of coordination (Shanahan, 2010; Tognoli and Kelso, 2014; Kasatkin et al., 2019; Venkadesh et al., 2020; Figure 1E). Spatiotemporal recordings of neural activity obtained from EEG and MEG, owing to their resolvability of high frequency oscillations such as gamma frequency bands (Figure 1B), allow for an interpretation of mesoscopic transitory dynamics using the framework of metastability (Freeman and Holmes, 2005). However, such a description might not be straightforward with the BOLD signals measured in fMRI because of its limitation in temporal resolution and precision of recorded neural activity. Hemodynamic responses, which follow the activation of local neural populations, are delayed by a few seconds due to the underlying neurovascular coupling mechanisms (Menon and Crottaz-Herbette, 2005). In addition, different brain regions may have different hemodynamic response profiles (Handwerker et al., 2004), making it challenging to identify spatially distributed regions that may be oscillating in a perfectly synchronized or phase-locked manner.

Another widely studied phenomenon that is applicable to systems of many interacting elements is self-organized criticality (SOC). It is hypothesized that the brain operates near the edge of criticality poised between total order and disorder (Werner, 2007; Hesse and Gross, 2014; Ma et al., 2019). The occurrence of mass events (e.g., temporal clusters of spikes in spatially distributed neurons) in a critical system exhibit power law distributions indicating spatiotemporal scale invariance. The power law distribution (or any deviations from it) empirically observed in neural ensembles is a way to characterize the critical nature of the spontaneous cortical dynamics (Miller et al., 2009; Petermann et al., 2009; Ma et al., 2019). However, it is also recognized that power laws alone may not be sufficient criteria for criticality (Beggs and Timme, 2012). Because abrupt transitions in collective dynamics are characteristics of neural networks operating near criticality (Werner, 2007), it is also possible that SOC may facilitate the spontaneous transitions between attractor states observed in metastable systems (Tognoli and Kelso, 2014). See Werner (2007) for an extensive review of various theoretical frameworks including SOC and metastability that fall under the umbrella of non-linear dynamics. The SOC is realized in a system only under certain conditions (Jensen and Magnasco, 1999), and the structural determinants of SOC have been investigated in a biologically plausible neural network model of spontaneous dynamics (Rubinov et al., 2011). It was shown that modular network structures with low-wiring costs, in the presence of activity-dependent synaptic plasticity, enhanced the regime for SOC. Modeling studies have also reported the significance of inhibitory synaptic plasticity in stabilizing networks near criticality (Stepp et al., 2015; Ma et al., 2019). Similarly, homeostatic structural plasticity can alter axonal and dendritic outgrowth to realize network criticality (van Ooyen and Butz-Ostendorf, 2019). These studies suggest that SOC may govern the network microstructural changes mediated through various plasticity mechanisms acting on multiple timescales.

Integrated information theory (IIT) postulates the properties of conscious physical systems that can account for phenomenological experience (Tononi et al., 2016). A moment of conscious experience is intrinsically *irreducible* to its distinct spatial features (integration), and it *differs* from all other realizable experiences (differentiation). The IIT defines complexity measures that quantify to what extent a network of elements is both integrated and differentiated (Oizumi et al., 2014). In its version 3.0, the IIT defines metrics such as cause-effect information, which measures the *specificity* of a mechanism in a certain state in constraining the system’s past and future states, and integration, which measures the *irreducibility* of the information generated by the whole system to the information generated by its parts. A network structure is partitioned into all possible candidate subnetworks, and their elements are perturbed into all possible states to identify local maxima of information integration. Thus, the rules governing the network structure-dynamics relationship are implicitly exploited in IIT’s quantitative framework. Moreover, IIT postulates that the global maximum of integrated information is specified at a *definite* spatiotemporal resolution in conscious physical systems. It was shown that such global maximum can occur at a coarser-grained level in space (grouped network elements) and time (grouped timesteps) in simple systems (Hoel et al., 2016). While IIT attempts to comprehensively characterize the necessary properties of conscious physical systems, application of its methods is limited to small and simple systems due to combinatorial complexities. It was also suggested that the temporal resolution at which the information integration reaches a maximum in the brain would correspond to the timescale of metastable attractors (Tononi, 2012). In a separate line of inquiry, it was hypothesized that a moment of conscious experience is intrinsically *irresolvable* in time, and the transitions between metastable attractor states in the brain coincide with moment-to-moment changes in conscious experience (Freeman, 1999). In the context of brain structure-dynamics relationship, it is worth pointing out that these frameworks treat consciousness as not a function of the brain, but a phenomenon that coexists with the dynamics of the brain. Finally, the mathematical relationship between integrated information and metastability has been examined in systems of coupled oscillators (Mediano et al., 2016), and this topic remains as a promising direction for future investigations.

## Macroscopic Relationships Between Structural and Functional Connectivity

The Default Mode Network (DMN) (Raichle et al., 2001) is one of the functional networks identified from PET and resting-state fMRI that consists of a number of anatomical hub regions such as the precuneus and posterior cingulate cortex. These hub regions have been suggested to be involved in self-referential processing (Northoff and Bermpohl, 2004; Cavanna and Trimble, 2006). Hagmann et al. (2008) reported a high degree of correspondence between the structural and functional connectivity of these regions in human brains. Shen et al. (2015) reported similar findings in macaque brains. Furthermore, these anatomical hub regions also showed higher degrees of synchronized activations in a whole-brain model conditioned on the dMRI connectivity (Schmidt et al., 2015). These studies highlight a strong link between the network topology and emergent functional patterns and suggest the role of anatomical hubs in functional integration.

The agreement between macroscopic structural connectivity and the functional connectivity that is estimated from low-frequency fluctuations has also been increasingly recognized. In a large-scale neural network model of the macaque cortex (Honey et al., 2007), intermittent synchronization was observed between regions on a (fast) time scale of hundreds of milliseconds. However, at the slowest time scale of minutes, the average of functional connections showed agreement with the underlying structural connections. Similar results showing agreement between structural and functional connections (only) on slow time scales were also reported in systems of coupled non-linear maps (Rubinov et al., 2009). Shen et al. (2015) empirically examined the influence of the anatomical architecture on the resting-state fMRI connectivity of macaque brains. They observed increasing similarity between structural and functional connectivity, when the size of the window to estimate functional connectivity was increased. Their results suggested that the functional connectivity estimated from the lowest frequencies of BOLD fluctuations best reflects the underlying structural connectivity.

Plasticity in the brain has been empirically observed in multiple timescales. Restoration of neuronal activations, following a lesion or monocular deprivation, can occur on a much longer timescale than the synaptic plasticity (Butz-Ostendorf and van Ooyen, 2017). Note that the Hebbian synaptic plasticity (Hebb, 2005) and synaptic scaling (Turrigiano et al., 1998) only adjust the strengths of *existing* synapses, and they facilitate activity restoration on a timescale of hours to a few days (Hengen et al., 2013; Barnes et al., 2015). However, other forms of structural plasticity that *rewire* synaptic connections (see section “Introduction”) can take place over weeks (Brown et al., 2009) or months (Giannikopoulos and Eysel, 2006) to restore circuit functions following lesions. Such structural adaptations can be viewed as brain’s compensatory responses to circuit damage. Thus, the relationship between brain structure and dynamics is governed by the mechanisms taking place over multiple timescales. Animal models are also useful in elucidating the plasticity mechanisms in the brain. In a mouse model of PD, a month of intense treadmill exercise was shown to increase dendritic spines and arborization in the striatum medium spine neurons, thereby reversing their loss of structural connections (Toy et al., 2014). Another study combined histological analysis with dMRI to examine structural plasticity induced by learning and memory in rats (Blumenfeld-Katzir et al., 2011). Their study reported increased fractional anisotropy in dMRI that was accompanied by an increase in the Myelin basic protein expression in certain regions, suggesting a neuronal basis for the white matter changes observed in dMRI that may be useful for indirectly localizing synaptic plasticity. It is worth noting that animal models allow experimental manipulations that are not possible in humans, and they can be valuable tools for translational research by identifying early biomarkers of neurological disorders (Gorges et al., 2017; Muñoz-Moreno et al., 2018).

Combined analyses of macroscopic structural and functional connectivity can reveal useful information about their relationships in pathological conditions. The structural connectivity enables a range of dynamics in the brain, and this has implications in neurodegenerative disorders such as Multiple Sclerosis (MS) and PD. A healthy brain exhibits a rich repertoire of dynamical configurations, which is revealed in the low similarity between structural and functional connectivity (Braun et al., 2015). At the onset of MS, the similarity between structural and functional connectivity is low, but as the disease progresses disconnecting structural pathways, they become highly similar, indicating reduced range of dynamical configurations (van Dam et al., 2021). Persson et al. (2006) investigated cognitive decline in aging and reported structural and functional alterations associated with declining memory performance in older subjects. The dMRI of these subjects showed reduced fractional anisotropy in anterior corpus callosum, and the fMRI showed increased activation in left prefrontal cortex. Increased activation in right prefrontal cortex was also reported for the subjects with the greatest memory decline. However, whether such increased activations are neural compensatory responses to the structural alterations is not clear, and models mechanistically integrating brain structure and dynamics (see section “Mechanistic Models of Brain Structure-Dynamics Relationship”) can reveal insights in this regard. Similarly, Poston et al. (2016) investigated neural compensatory mechanisms in PD, where the loss of dopamine neurons in basal ganglia is believed to be at least partially responsible for the motor symptoms. Despite severe neurodegeneration in the basal ganglia, many PD patients remain cognitively intact. Poston et al. (2016) reported increased activation of the putamen in the fMRI of cognitively intact PD patients compared to the healthy group during working memory tasks. Their study suggested that the hyperactivation of the putamen is a compensatory response to the loss of dopamine neurons to maintain normal cognition in PD. Such neural compensatory mechanisms are bound by the structure-dynamics relationship of the brain.

## Mechanistic Models of Brain Structure-Dynamics Relationship

The synaptic plasticity mechanisms that mediate structure-dynamics coupling at the micro- and mesoscopic level are largely elusive in the connectivity estimated from neuroimaging modalities such as diffusion-weighted and functional MRI [although see Blumenfeld-Katzir et al. (2011)]. Thus, the mesoscopic dynamics of neural ensembles are of special interest to investigate the reciprocal relationship between the network structure and dynamics. Models of whole-brain oscillatory dynamics aim to simulate multiscale mechanisms (Schmidt et al., 2018; Shen et al., 2019) and enable perturbational methods of inquiries (Deco et al., 2015) to probe into their relationships. However, achieving the right balance between abstraction and biophysical details to describe various elements of neural networks is challenging, and it is an important methodological consideration in large-scale models of brain dynamics.

Large-scale neural network simulations aiming to capture biophysical details in a comprehensive manner are computationally demanding. Such simulations typically describe rules governing various neuronal ion-channel kinetics to account for the electrophysiological diversity of neuronal populations (Morgan and Soltesz, 2008; Markram et al., 2015; Bezaire et al., 2016). Morphologically detailed Hodgkin-Huxley type models of individual neurons (Hodgkin and Huxley, 1952) specify hundreds of differential equations for each neuron limiting the scalability of network simulations. On the other hand, simpler phenomenological models such as the Quadratic Integrate and Fire (Izhikevich, 2003) and Adaptive Exponential Integrate and Fire (Naud et al., 2008) have been recently shown to quantitatively capture the diversity of neural excitability patterns using powerful optimization techniques (Teeter et al., 2018; Venkadesh et al., 2019). These simpler neuronal models can significantly reduce the computational demands of large-scale network simulations (Izhikevich and Edelman, 2008).

Model reduction techniques for the network dynamics have also been proposed. One approach to simplify the network model complexity is via dynamic mean-field (DMF) reduction (Wong and Wang, 2006). Here, neural population dynamics are reduced to single units that approximate the input-output relation (e.g., current – frequency curves) of the population. Such generalizations significantly reduce the number of differential equations that describe the temporal evolution of the model. It is worth noting that the DMF techniques that capture population firing frequencies in single unit approximations do not account for the emergent self-organizing patterns of interacting neurons. For example, selectively recruited individual pyramidal neurons in the CA1 and CA3 area of the rodent hippocampus not only collectively fire in theta-modulated-gamma frequencies, but their firing occurs at *different phases* of the background theta oscillations, correlating with the location of the animal in an environment (O’Keefe and Recce, 1993; Dragoi and Buzsáki, 2006). These temporal features underscore the dynamical complexity at the level of individual neurons. Such temporal structures emerge from neuronal interactions mediated by the microcircuit connectivity and single-unit approximations of population dynamics lack explanatory power at this level. However, DMF models constrained by macroscopic- neuroanatomical connectivity showed agreement with the resting state functional connectivity that was viewed as a static map of interactions between regions (Deco et al., 2013; van Hartevelt et al., 2014).

Several computational studies have investigated the relationship between empirically measured structural and resting-state functional connectivity by simulating large-scale network dynamics. Population dynamics of local brain regions can be described by mutually interacting excitatory pyramidal-type neurons and inhibitory interneurons, and the connections between (pyramidal neurons of) the brain regions can be specified using the empirically measured structural connectivity (Deco and Jirsa, 2012; Deco et al., 2013). It is worth mentioning that the most prominent inhibitory interneurons such as the Parvalbumin positive Basket cells typically have higher frequency profiles than the pyramidal neurons. The differences in their intrinsic excitability could induce bursting dynamics when they mutually interact (Izhikevich, 2007), and bursting dynamics maybe crucial to realize metastable complexity in neural networks (Venkadesh et al., 2020) (see section “Frameworks to Characterize Network Dynamics” for a discussion on metastability). This suggests an importance of capturing mesoscopic neural diversity in large-scale network simulations.

Alternative to the DMF techniques, coupled phase oscillators such as Kuramoto models have also been used to reduce the network model complexity. Here, oscillators represent brain regions, and they are coupled according to the cerebral connectome to investigate the emergent synchronization patterns. Using Kuramoto oscillators, Schmidt et al. (2015) showed that the high-degree hub regions in the brain anatomical connectivity (van den Heuvel and Sporns, 2011) synchronized at a higher level than other regions and were also crucial in achieving intermodular synchronization. Similarly, pulse-coupled phase oscillators have been simulated using a fully-connected network with synaptic plasticity (Kasatkin et al., 2019). Here, oscillators self-organized into multiple volatile domains of synchronized and desynchronized activity called itinerant chimeras, a special case of metastability. More importantly, the plasticity rules enabled the emergence of positive structural connections within the coherent domains and negative connections across domains, highlighting an interaction between the network structure and the itinerant/metastable dynamics. Similarly, Rubinov et al. (2009) simulated spontaneous cortical dynamics on structural connectivity networks with activity-dependent plasticity rules. Not only did modular functional patterns emerged from initial structural networks with random connectivity, but the connectivity was also rewired toward more modular structures corresponding to the functional patterns.

The resting-state fMRI paradigm provides additional empirical ground to fit the simulated large-scale oscillatory dynamics to the correlations in the BOLD activity between brain regions. As mentioned before, BOLD signals represent regional activations convolved by low frequency hemodynamic responses. A hemodynamic model (Friston et al., 2003) can be specified to obtain simulated BOLD activity from the population firing rates of neurons. This approach revealed that the time-averaged resting-state functional connectivity emerged from slow and stable linear firing activity close to a point of bifurcation (Deco et al., 2013). A bifurcation is a qualitative change in a dynamical system behavior and such bifurcation points are identified by tuning a global coupling constant (bifurcation parameter). Taking a similar approach, van Hartevelt et al. (2014) reported the effects of long-term deep brain stimulation (DBS) of subthalamic nuclei on structural and functional connectivity of a Parkinson’s disease patient. Following 5 months of DBS, the dMRI showed no notable change in global graph metrics, but significant differences in local graph metrics for several regions were observed. Moreover, the post-DBS structural connectivity shifted the global bifurcation of the simulated functional connectivity toward the healthy bifurcation. Such whole-brain computational models of spontaneous dynamics are valuable tools to study the effects of local alterations in structural connectivity on the global brain dynamics. However, a limitation of such approaches is that they do not take into account the dynamical topographic organizations of the resting-state functional connectivity (Betzel et al., 2016). Future whole-brain modeling studies should attempt to capture such time-varying organizations in simulated functional connectivity. Such models can potentially reveal novel biomarkers for neurological disorders (Figure 1F) based on the transient network dynamics (Lord et al., 2017).

## Discussion

At the mesoscopic level of neuron types, frameworks relating neural diversity and emergent network complexity are in urgent need of development. While considerable progress has been made in characterizing different *types* of elements of neural networks, how these diverse elements coordinate with each other in a way that allows for the emergence of coherent dynamics is still an open question. Comprehensively mapping the many-to-one relationships between structural network configurations of diverse neural elements and their characteristic dynamical complexities in the nervous system is an important milestone to achieve. This will require formulating scalable mathematical characterizations of brain dynamics and identifying distinct combinations of diverse neural elements that could realize a precise dynamic given a connectivity structure. This could be formulated as a search problem in the space of parameters describing neural diversity and connectivity. Powerful optimization techniques, such as Evolutionary Algorithms (EA) (De Jong, 2016), can apply selection pressure to these parameters using a fitness function describing a dynamical complexity. By appropriately configuring the exploration and exploitation tendencies, the EAs can, in principle, identify multiple structural configurations that could realize the given dynamical complexity. GPU-accelerated simulations of network dynamics (Nowotny et al., 2014; Beyeler et al., 2015) will also be integral to such computationally demanding objectives. Such frameworks can make predictions about the *optimal* combinations of diverse neural elements in a network, which can be validated on large-scale datasets that quantitatively characterize mesoscopic level neural diversity and connectivity (Wheeler et al., 2015; Teeter et al., 2018; Komendantov et al., 2019).

The macroscopic anatomical connectivity estimated from dMRI is based upon the diffusion of water along axonal fibers. The fiber orientation at each voxel is estimated by calculating orientation distribution function, which is a distribution of water diffusion at various orientations. Techniques that rely solely on the local orientation information could result in false-positive connections (Maier-Hein et al., 2017). Approaches that supplement this local orientation information with a global model of diffusion signals could improve the tractography results. Streamline filtering based on spherical-deconvolution (Smith et al., 2013) and combining microstructural features with tractography (Daducci et al., 2015) are some of the approaches that specify such global models, which are solved using powerful optimization techniques. Note that the streamline/fiber counts estimated from dMRI tractography are typically assumed to represent the *strength* of connections between regions in connectivity analyses. However, the fractional anisotropy, which is used in tractography, is sensitive to features such as the degree of myelination and curvature in axonal pathways (Jones et al., 2013). Therefore, streamline counts estimated from dMRI may only approximate the actual fiber counts between brain regions. Moreover, the choice of the tractography technique and its parameters can influence the computed graph metrics. Duda et al. (2014) evaluated the reproducibility of graph metrics using different tractography algorithms and their sensitivities to different network sparsity levels. While global and local efficiencies (see section “Characteristic Measures of Network Connectivity”) were highly reproducible across fiber tracking algorithms and network sparsity levels, characteristic path length and rich club coefficient were sensitive to the choice of fiber tracking algorithm. Their study also suggested the importance of computing these metrics over a range of network sparsity thresholds (and degree thresholds for rich-club coefficient).

Obtaining comprehensive measurements of neural activation patterns that cover *both* the breadth (i.e., whole-brain coverage) and the depth (i.e., at a sufficient spatiotemporal resolution) of a human brain remains technologically challenging. While EEG and MEG can record neural activations at a high temporal resolution, they can only capture activations from limited types of neurons. They can measure activities of cortical pyramidal neurons, which have morphologies in a laminar organization. Such neuronal architectures enable dipole fields from populations of neurons to be detected at the scalp. However, cortical interneurons that lack laminar organization might not contribute significantly to the EEG or MEG signals, if their dendrites and axons spatially overlap, canceling out strong electromagnetic fields (Freeman et al., 2009). Such structural features also make detecting neuronal sources of activations in deeper brain regions by EEG and MEG difficult, although recent efforts (Pizzo et al., 2019) show promise in this regard. fMRI BOLD signal measurements, on the other hand, rely on the hemodynamic responses to neuronal activations and are not sensitive to neuronal structural organizations, thereby enabling more spatially comprehensive measurements of their activations. However, the BOLD signal measurements are influenced by the underlying neurovascular coupling mechanisms (Menon and Crottaz-Herbette, 2005). The coupling between neuronal activation and subsequent hemodynamic response involves complex pathways that include astrocytes (Wolf and Kirchhoff, 2008) and neurotransmitters such as glutamate and GABA (Muthukumaraswamy et al., 2012). Understanding the mechanisms of neurovascular coupling and incorporating those mechanisms in models of hemodynamic responses (Hillman, 2014; Huneau et al., 2015) are necessary to appropriately interpret the lagged and negative correlations observed in fMRI time series. The complementary nature of functional imaging modalities EEG/MEG and fMRI maybe valuable to improve the spatiotemporal resolution of recorded brain activity through cross-modal integration (Freeman et al., 2009; Hall et al., 2014; Cichy and Oliva, 2020). Such multimodal integration is beneficial, for example, to identify the frequency bands of neuronal activations that are reflected in hemodynamic responses (Uono et al., 2017) and to improve the predictive accuracies of biomarkers based on functional neuroimaging (Engemann et al., 2020).

The mechanistic relationship between brain structure and dynamics is mutual. While structural connectivity constrains the range of emergent dynamics, the dynamics, in turn, shapes the network microstructure on multiple timescales to achieve optimal function. For instance, electrical activity of neurons modifies synaptic strengths on short timescales mediated by Hebbian plasticity (Hebb, 2005), and modifies neuronal morphology on much longer timescales mediated by intracellular calcium concentration (Butz and van Ooyen, 2013) (see also section “Macroscopic Relationships Between Structural and Functional Connectivity”). However, the principles of interactions between various plasticity mechanisms acting across timescales are unknown. The top-down influence of collective neuronal dynamics, under the constraints of anatomical connectivity, likely regulates such interactions (see also discussion on SOC in section “Frameworks to Characterize Network Dynamics”). Therefore, quantitative frameworks characterizing multiscale dynamical complexities in neural networks are of utmost importance. Such frameworks, when numerically scalable, will enable interpretation and validation of empirically observed structure-dynamics relationships.

Finally, the structural human connectome is incomplete without the descriptions of neural connections in the spinal cord and peripheral nervous system (Irimia and Van Horn, 2021). The structure-function relationship in the nervous system, in its broadest scope, concerns itself with an important question: what are the mechanisms by which the whole-organism connectomes realize autonomy in their actions? (see Albantakis et al. (2020) for a discussion of autonomy) Neural constituents of these mechanisms are shaped by the environmental inputs, which are integrated into the brain dynamics via action and perception (Freeman and Holmes, 2005). Delineating the multifaceted relationship between neural structures and their intrinsic dynamics in the brain is a necessary step toward a foundational understanding of nervous system functions in normal and aberrant conditions.

## Author Contributions

SV and JV contributed to the conception of this work and manuscript revision. SV wrote the first draft of the manuscript. Both authors approved the submitted version.

## Conflict of Interest

The authors declare that the research was conducted in the absence of any commercial or financial relationships that could be construed as a potential conflict of interest.

## Publisher’s Note

All claims expressed in this article are solely those of the authors and do not necessarily represent those of their affiliated organizations, or those of the publisher, the editors and the reviewers. Any product that may be evaluated in this article, or claim that may be made by its manufacturer, is not guaranteed or endorsed by the publisher.
